# An alcohol-related liver disease multi-stakeholder hub (ARMS-Hub) to enhance research activity in underserved communities in the UK

**DOI:** 10.3310/nihropenres.13598.2

**Published:** 2024-10-29

**Authors:** Ashwin Dhanda, Victoria Allgar, Neeraj Bhala, Lynne Callaghan, Joana Castro, Shilpa Chokshi, Amanda Clements, Wendy Clyne, Colin Drummond, Ewan Forrest, Lesley Manning, Richard Parker, Debbie Shawcross, Jennifer Towey

**Affiliations:** 1 Faculty of Health, University of Plymouth, Plymouth, England, UK; 2 South West Liver Unit, University Hospitals Plymouth NHS Trust, Plymouth, UK; 3 School of Medicine, University of Nottingham, Nottingham, England, UK; 4 Roger Williams Institute of Hepatology - Foundation for Liver Research, London, UK; 5 Faculty of Life Sciences and Medicine, King's College London, London, England, UK; 6 Institute of Psychiatry, King's College London, London, England, UK; 7 School of Cardiovascular and Metabolic Health, University of Glasgow, Glasgow, Scotland, UK; 8 Public member, Plymouth, UK; 9 Department of Hepatology, Leeds Teaching Hospital NHS Trust, Leeds, UK; 10 Therapy Services, University Hospitals Birmingham NHS Foundation Trust, Birmingham, UK

**Keywords:** alcohol-related liver disease, ArLD, liver disease, stigma, PPIE, people with lived experience, mental health

## Abstract

**Background:**

Alcohol use is the third leading risk factor of death and disability in the UK and costs the NHS £3.5 billion per year. Despite the high prevalence and healthcare burden of Alcohol-related Liver Disease (ArLD), there has been minimal research addressing prevention, morbidity and mortality. Reasons for this include stigma and lack of interest from public, charitable and commercial funding bodies. The objectives of this project were to identify and develop interdisciplinary partnerships, to explore stigma in ArLD, to develop a representative Patient and Public Involvement and Engagement (PPIE) group, to build research capacity, and to develop interdisciplinary research proposals targeting key research priorities.

**Methods:**

ArLD networks were identified by members of the Project Steering Group. Health Care Professionals (HCPs) from different backgrounds were invited to join the ARMS-Hub. PPIE representatives were invited through charities and support groups. Research areas were identified, discussed, prioritised and ranked. Research questions were refined during an in-person symposium. A mentorship programme was created to encourage and facilitate networking and knowledge exchange for early career researchers.

**Results:**

We established the ARMS-Hub with 31 HCPs and 40 PPIE members. There were five stakeholder meetings, which included PPIE representation. Three virtual and three in-person PPIE meetings took place. Topics relevant to stigma in ARLD identified during the meetings were education and awareness, language, and access. Priorities identified were the disconnect between mental health and liver services, education around the wider harms of alcohol, and education of HCPs regarding stigma. We established a mentorship network that regularly meets to support development of new research ideas.

**Conclusions:**

Stigma is central to lack of research engagement from professionals and PPIE. The main priority identified relates to the disconnect between mental health and liver services. This collaborative study has allowed development of a research agenda to address this priority.

## Plain language summary

**Background:**

Alcohol-related liver disease (ArLD) is a big problem and the third most common cause of death and disability in the UK. Alcohol use costs the NHS billions each year. However, partly due to the stigma associated with ArLD, research into preventing, treating, and understanding it has been limited.

**Aim:**

We aimed to create a network of ArLD experts and people with lived experience and their carers (ARMS-Hub). By working together, we aimed to improve research on ArLD through understanding what research is needed and how best to carry it out.

**Methods:**

We identified existing ArLD networks and invited health workers (doctors, nurses, allied health professionals), scientists, academics, people from the voluntary/ charity sector, and people with lived experience and their carers to join our hub. We talked about stigma and priorities for ArLD research in a series of online and in-person meetings. A mentorship network was also created to help share information and develop research capacity.

**Results:**

Thirty-one health workers and 40 people with lived experience or their carers attended 12 ARMS-Hub meetings. Discussions focused on education of the public and health workers on the harms of alcohol, the use of stigmatising language and complex medical terms, access to liver and mental health services and the culture around alcohol use. The areas considered most important to the group for future research were:

- Integration of mental health and liver services;
- education on effects of alcohol use;
- training of health workers on alcohol stigma.

**Dissemination:**

In the final meeting, we worked on a proposal for a research project to improve the recognition and treatment of mental health problems in people attending hospital liver clinics.

## 1. Introduction

Alcohol use is the third leading risk factor of death and disability in the UK, the leading cause of death in working age men, and is the main driver of chronic liver disease, estimated to affect 600,000 people in England alone ^1^. Alcohol use costs the NHS £3.5 billion per year ^2^. There were 27,419 hospital admissions and 7,518 deaths specifically due to Alcohol-related Liver Disease (ArLD) in 2022 in the UK ^3^. Inpatient mortality is as high as 23% and many of these were first presentations of advanced ArLD cirrhosis indicating low rates of early identification and intervention ^4^.

In 2014, the Lancet Liver Commission recognised harmful alcohol use as the main driver of liver disease that needs to be urgently addressed ^1^. In 2017, the James Lind Alliance conducted a priority setting partnership to identify the top ArLD research questions ^5^. Unfortunately since then little progress has been made in research and health policy and ArLD admissions and mortality continue to rise year on year.

ArLD is a condition that is driven by major health inequalities. People in deprived communities are ten times more likely to develop ArLD and subsequently die from it than the most affluent ^6^. There has also been increasing incidence of ArLD in ethnic minorities and women, with disparities in access to alcohol treatment ^1^.

Despite the high prevalence and healthcare burden of ArLD, there has been a paucity of research addressing prevention, morbidity and mortality, and treatment of alcohol use disorder underlying it. International data assessing research activity and liver disease burden found a marked inattention to ArLD compared to other liver diseases ^7^.

Reasons for inattention to ArLD are multifactorial but certainly include stigma and reduced appetite for research funding from public, charitable and industry bodies. Stigma impacts healthcare of ArLD patients at all stages of disease from early detection to intervention ^8, 9^. Public stigma that ArLD is self-inflicted may influence allocation of industry and non-commercial funding. A recent stigma survey undertaken by the British Liver Trust found that over 70% of people with liver disease had personally experienced stigma and 49% had experienced it from Healthcare Professionals (HCPs). NHS Scotland has made the first steps to change public opinion by challenging stigma around alcohol use ^10^. Wider engagement of public, HCPs and industry is required to impact the stigma associated with harmful alcohol use and its consequences.

Furthermore, ArLD is a complex condition requiring an integrated multidisciplinary approach including hepatologists, addiction psychiatrists, alcohol nurses, allied health professionals and community workers. Current research mainly occurs in specialty silos and fails to draw together the multidisciplinary team needed to conduct successful ArLD research. There are large gaps in ArLD research including qualitative research, research in non-hospital settings, participation of non-medical HCPs and involvement of community services and the voluntary sector. This helps to explain the lack of progress in answering top research priorities such as methods to deliver healthcare education about excessive alcohol use and integrated community-based care models.

There is an urgent unmet need to increase and enhance ArLD research in the UK. This project was an opportunity to explore stigma and develop methods to challenge it and to increase engagement in areas of high prevalence and low research activity. It created a multidisciplinary partnership across different settings that contributed to develop a research proposal addressing the most important research priority to improve care and treatment and thus reduce morbidity and mortality from ArLD.

### 1.1. Aims and objectives

The aim of the project was to establish an ArLD multi-stakeholder hub (ARMS-Hub) of multidisciplinary experts and patient and public involvement and engagement (PPIE) representatives that will increase and enhance ArLD research in the UK through identification of research priorities. The objectives were:

1. To identify and develop interdisciplinary partnerships including community, voluntary sector and social care organisations.
2. To explore stigma in ArLD research and methods to overcome it.
3. To develop a diverse and representative ArLD PPIE group.
4. To build research capacity by engaging less research-active clinicians, non-medical healthcare professionals and community workers.
5. To develop interdisciplinary research proposals targeting key research priorities to enhance ArLD research in the UK in preparation for a future grant submission.

## 2. Methods

### 2.1. Creation of ARMS-Hub

The Project Steering Group was created by the Chief Investigator using his professional network and ensuring representation of relevant professions and disciplines. It formed the backbone of the ARMS-Hub and consisted of a person with lived experience, five hepatologists, a nurse consultant, a dietitian, an addiction psychiatrist, a methodologist/statistician, a Research Design Service director and a qualitative researcher. We built on this interdisciplinary expertise by inviting a diverse group of stakeholders ranging from senior doctors and nurses to scientists, academics, allied health professionals (AHPs), industry representatives and people from the voluntary and charity sector. They were identified and invited to join the ARMS-Hub through the mapping of research and clinical networks and through contacts and professional networks from the members of the Project Steering Group across disciplines and settings. A list of relevant organisations and individuals was created by the group. Each individual or a representative from each organisation was contacted by email by a member of the Project Steering Group and provided with information about the project and its objectives. If no response was received, a further reminder email was sent on one occasion. Recruitment did not include identification or approach of stakeholders through NHS or adult social care services. We invited representatives from the British Association for the Study of the Liver ArLD Special Interest Group, Royal College of Psychiatrists Addiction Faculty, British Liver Nursing Association, Society for the Study of Addiction, Alcohol Health Alliance, British Dietetic Association, Chartered Society of Physiotherapy and Royal College of Occupational Therapists as well as the national charities Alcohol Change UK, National Association for Children of Alcoholics and Humankind.

Stakeholder meetings including PPIE representatives were conducted virtually. The first three ARMS-Hub meetings discussed the following issues: 1) ‘Is stigma still a problem in developing and conducting ArLD research and how can we overcome this?’; 2) methods to engage less research-active sites and non-medical healthcare professionals in ArLD research; 3) methods to engage under-served communities with ArLD in research. The fourth meeting generated and prioritised ArLD research questions (in the light of findings from earlier workshops on stigma and engagement). The final meeting was used to refine the highest priority research question.

Members of the Project Steering Group (Dhanda, Callaghan, Castro and Manning) facilitated meetings. Topics (stigma, inclusion and engagement) were discussed in virtual breakout rooms with attendees having the opportunity to contribute to each one. Facilitators gave prompts but topics were kept open so new ideas and perspectives could emerge. At the fourth meeting, an adapted nominal technique approach ^11^ was used to prioritise each research area until consensus was reached. Using this technique, facilitators ensured that all attendees were able to have an equal voice and contribute to the prioritisation. The fifth stakeholder meeting involved working in small groups on the three top priority areas to produce relevant questions to address each area. The top priority question was taken forward for dedicated work in the research symposium.

Attendees’ consent to collect and store their personal information (contact details, profession and employer) for the purpose of this research project was obtained when they registered to attend an ARMS-Hub meeting using an online form. Consent from PPIE representatives is described below. Virtual meetings were not recorded but were audio-transcribed using the meeting platform software. Facilitators also took notes. Data was analysed according to the themes that consistently appeared in each sub-group discussion.

Stakeholders were compensated for their time according to their pay band and PPIE members received retail shopping vouchers at a rate of £25 per hour.

### 2.2. Public and Patient Involvement and Engagement

To ensure representation and accessibility, we held both virtual and in-person meetings. Members were invited through the project’s PPIE co-applicant who identified nationwide charities and support groups. We also engaged with the British Liver Trust patient forum, a group of over 500 patients with liver disease. PPIE representatives for in-person meetings were invited at an alcohol support service in Plymouth to be able to include people without access to technologies and who tend to engage less in research. Recruitment did not include identification or approach of representatives through NHS or adult social care services.

Informed consent to collect and store attendees’ personal data was obtained before they took part in any meetings. For virtual meetings, PPIE representatives completed an online form providing information on their age range, gender, ethnicity, education status, liver disease, alcohol use and postcode (for calculation of deprivation status). For in-person meetings, attendees completed a paper form with the same data as the virtual form and were provided support from facilitators if needed. If they were attending as a carer or advocate of someone with ArLD, they were asked to provide information relating to the person with liver disease and not themselves. On both online and paper-based form, there was a box for attendees to tick to express their consent.

The Index of Multiple Deprivation (IMD) was worked out using the attendee’s postcode using open source data at https://imd-by-postcode.opendatacommunities.org/imd/2019. The IMD is a rank and is reported in deciles. The deciles are determined by categorising the areas nationwide based on their level of deprivation, and then dividing them into 10 equally sized groups. Decile 1 represents the 10% most deprived areas in England, whereas decile 10 represents the 10% least deprived. Measures of deprivation include income, access to employment, education, skills and training, healthcare, disability, crime, housing and services, and living environment.

### 2.3. Equality, Diversity and Inclusion

As people with ArLD come from different socioeconomic backgrounds and communities, there are some sub-groups that do not always engage with healthcare services and research. To ensure that a full range of ArLD patient voices contributed to the ARMS-Hub, we developed a strategy to ensure diverse representation. The strategy was supported by a designated Equality, Diversity and Inclusion (EDI) Lead (Bhala), who used his experience of understanding ethnic disparities in liver disease identification and outcomes to ensure inclusion of a diverse spectrum of people with ArLD. We identified and invited leaders from ethnic minority groups to join the ARMS-Hub and assist with recruiting PPIE representatives from their networks.

### 2.4. Research symposium

The wider group was divided into three sub-groups and worked on tackling the research question ‘ *Does integration of mental health services into liver services improve quality of life and clinical outcomes for people with alcohol-related liver disease?’* Everyone had the opportunity to contribute to the three proposed Work Packages (WPs): WP1) Evidence mapping and synthesis; WP2) Co-design of an integrated service; WP3) Feasibility of implementation and evaluation of service. Information was gathered on how to map existing services, methods to design an intervention and key stakeholders to involve, and what outcomes and measures to use.

### 2.5. Mentorship programme

To build research capacity, we aimed to connect early career researchers that wanted to upskill with experienced researchers who could share guidance and expertise. Initially the plan was to have a formal mentorship arrangement in which a mentee and mentor would be paired. However, after the first meeting, the preference of attendees was to keep the discussions open to the whole group as there was an array of expertise and knowledge that was more fruitful if shared in a mentorship network approach. Individuals were still able to meet separately with experts if they wished, to discuss specific projects or knowledge gaps. A long-lasting relationship was encouraged beyond the scope of this project. Interested parties are still able to access the scheme through the Hub website.

### 2.6. Ethical considerations

This project did not involve identifying patients through NHS or social care services and did not include them in research. Therefore, NHS Research Ethics Committee approval was not required. This was confirmed using the online NHS Health Research Authority decision tool ^12^. Responses for each UK nation are available in the supplementary material. Informed consent was obtained, as described in section 2.2, to collect personal data according to the Data Protection Act 2018. For stakeholders and early career researchers, this consent was obtained by asking for confirmation in the response to the invitation email that they agreed to share their name, organisation, profession or role and contact email with the study team. Personal data will be stored for five years after the completion of the project to enable the ARMS-Hub to continue to function even after funding ends.

The information shared in the discussions was transcribed straight from the virtual meeting platform using its in-built transcription feature. There was no video or audio recording. All data was anonymised, ensuring confidentiality, and presented in a way that does not allow traceability. Access was restricted to the Chief Investigator and Research Coordinator.

### 2.7 Data analysis

Categorical data (e.g. gender) is described using absolute numbers and percentage of total. Continuous data (e.g. ranking scores) is described using mean only. No statistical analysis has been conducted to compare any groups.

Information from meetings was summarized with hand-written notes. These were not formally analyzed using any qualitative framework as the meetings constituted PPIE and stakeholder discussions rather than qualitative research data. Our approach was in line with NIHR recommendations ^13^ and standards for reporting of PPIE ^14^. Notes were reviewed by researchers (Callaghan, Castro, Dhanda and Manning) after each meeting and main topics identified and agreed by consensus. The same process was undertaken for determining sub-topics from discussions.

## 3. Results

### 3.1. ARMS-Hub

Organisations were identified through mapping clinical and research networks nationwide and through contacts and professional networks of members of the Project Steering Group. Representativeness was ensured by inviting experts from diverse disciplines and settings including hepatology, addictions, biomedical science, community services and charities/voluntary sector. Organisations represented in the ARMS-Hub were The British Liver Trust, British Association for the Study of the Liver, British Society of Gastroenterology, British Association of Allied Health Professionals in Liver, British Liver Nurse Association, The Roger Williams Institute of Hepatology, CyberLiver, British Dietetic Association and the Gastro London Investigative Network for Trainees. Representatives from relevant national charities also participated in discussions, namely from Alcohol Health Alliance, the Royal College of Physicians, Guts UK charity and Humankind Charity.

Fifty-seven people registered their interest and 31 attended one or more of the five stakeholder meetings and the research symposium. The attendance rate was, on average, 11 people per session, ranging from four to 19. Each meeting had a mixture of doctors, nurses, AHPs and scientists, as well as representatives of the charity sector and the PPIE group.

### 3.2. PPIE in-person group

***3.2.1. Sociodemographic data.*** The PPIE in-person group consisted of 31 attendees. Where attendees were carers for someone living with Alcohol-related Liver Disease (ArLD), the answers relate to the patient and not the carer. Some questions weren’t answered or attendees preferred not to disclose the information. 

Most attendees were people living with ArLD (58%), 23% were carers and 19% accounts for missing data ( Table 1). They were mostly between 45–54 years old (29%), 55–64 years old (23%) and 35–44 years old (19%). 23% of the attendees were female, 68% were male and 9% didn’t answer. The majority was White British/ Irish (81%), while White (Other), Asian or Asian British and missing data accounted for 6% each.

**Table 1.** Sociodemographic and liver disease characteristics of the PPIE groups.

*Sociodemographic characteristics*	In-person group (n=31), number (%)	Virtual group (n=9), number (%)
**Age** 25 – 34 35–44 45–54 55–64 More than 64 Missing data/ Prefer not to say	4 (13) 6 (19) 9 (29) 7 (23) 1 (3) 4 (13)	1 (11) 1 (11) 4 (44) 2 (22) 0 1 (11)
**Education** None Primary school Secondary school College or University Undergraduate degree Post-graduate degree Missing data/ Prefer not to say	3 (10) 1 (3) 8 (26) 9 (29) 6 (19) 1 (3) 3 (10)	0 0 2 (22) 1 (11) 1 (11) 4 (44) 1 (11)
**Occupation** Unemployed or not working due to long-term sickness Skilled manual worker Intermediate managerial/ professional/ administrative Supervisory or clerical/ junior managerial/ professional/ administrative Carer of other household member Higher managerial/ professional/ administrative Student Retired Missing data/ Prefer not to say	20 (64) 2 (6) 0 1 (3) 1 (3) 0 1 (3) 0 6 (19)	1 (11) 0 5 (56) 1 (11) 0 1 (11) 0 1 (11) 0
***Liver Disease***		
**Liver Disease status** Alcohol-related fatty liver disease Fibrosis Cirrhosis Liver failure Hepatitis C Missing data/ Prefer not to say	10 (32) 3 (10) 2 (6) 10 (32) 1 (3) 15 (48)	1 (11) 1 (11) 1 (11) 3 (33) 0 3 (33)
**Current drinking habits** Yes No Missing data/ Prefer not to say	13 (42) 17 (55) 1 (3)	1 (11) 8 (89) 0
**Average of units of alcohol *per* week** 1–14 15–34 35–50 50 or more	2 (15) 5 (38) 2 (15) 4 (31)	0 1 (11) 0 0

In terms of education, 29% went to College or University, 26% completed secondary school, and 19% completed an undergraduate degree ( Table 1). Regarding occupation, 64% were unemployed or not working due to long-term sickness and 19% didn’t answer ( Table 1).

**Figure 1.**
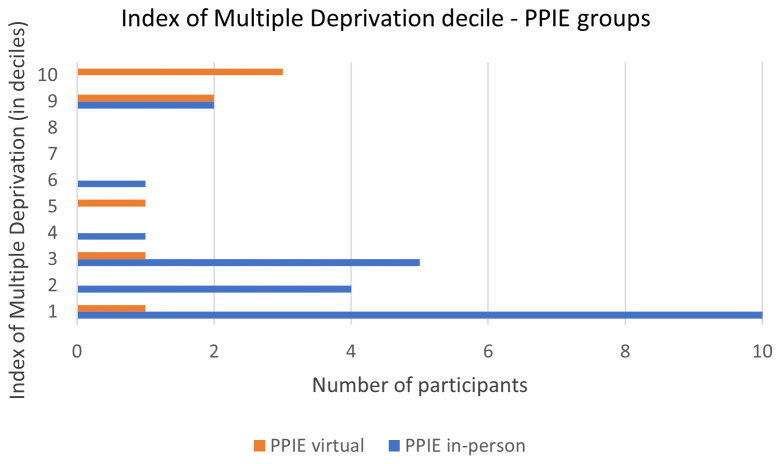
Deciles of index of multiple deprivation of the virtual and in-person PPIE groups. Decile 1 is most deprived and decile 10, least deprived.

***3.2.2. Deprivation index.*** Index of Multiple Deprivation (IMD) was obtained via the postcode, which 23 attendees provided. The majority of attendees (65%) live in the most deprived areas of England (deciles 1 to 4), with 32% living in the 10% most deprived areas in the country (decile 1; Figure 1).

***3.2.3. Liver Disease and current drinking habits.*** Self-reported liver disease status included alcohol-related fatty liver disease (32%; now renamed as steatotic liver disease to be less stigmatising) ^15^ and liver failure (32%), although some attendees selected more than one answer. 48% chose not to answer.

55% of the attendees did not drink alcohol at the time of answering the questionnaire. Three attendees did not answer the question clearly and their data were therefore excluded. For those who reported current drinking habits, 38% reported drinking 15–34 units per week, 31% drank 50 units or more, and 15% drank 1–14 and 15% 35–50 units per week.

### 3.3. PPIE virtual group

***3.3.1. Sociodemographic data.*** Fourteen people registered their interest in attending the virtual PPIE meetings and nine participated in one or more meetings out of the three PPIE meetings and the research symposium. On average, four people attended each meeting, ranging from three to five attendees.

Five attendees were people living with ArLD and four were carers ( Table 1). Four attendees were 45–54 years old, two were 55–64 years old and one each were 25–34 and 35–44 years old. Six attendees were male. In terms of ethnicity, eight were White British/ Irish and one was Asian or Asian British.

Regarding education, four were educated at post-graduate level, while two completed secondary school, one College or University and one undergraduate degree. Five of the attendees worked at intermediate managerial/ professional/ administrative positions, one was unemployed or not working due to long-term sickness, and one each worked at supervisory or clerical/junior managerial/professional/administrative roles, higher managerial/professional/administrative or were retired.

***3.3.2. Deprivation index.*** Eight of the nine attendees provided their postcode. Most of them (5 out of 8) live in the least deprived areas of England (deciles 9 and 10; Figure 1).

***3.3.3. Liver Disease and current drinking habits.*** In terms of Liver Disease status, three had liver failure, one each suffered from alcohol-related steatotic liver disease, fibrosis or cirrhosis. The remaining three are missing data or chose not to answer. Eight did not currently drink alcohol while one reported drinking 15–34 units of alcohol per week.

### 3.4. Stakeholder group

**Figure 2.**
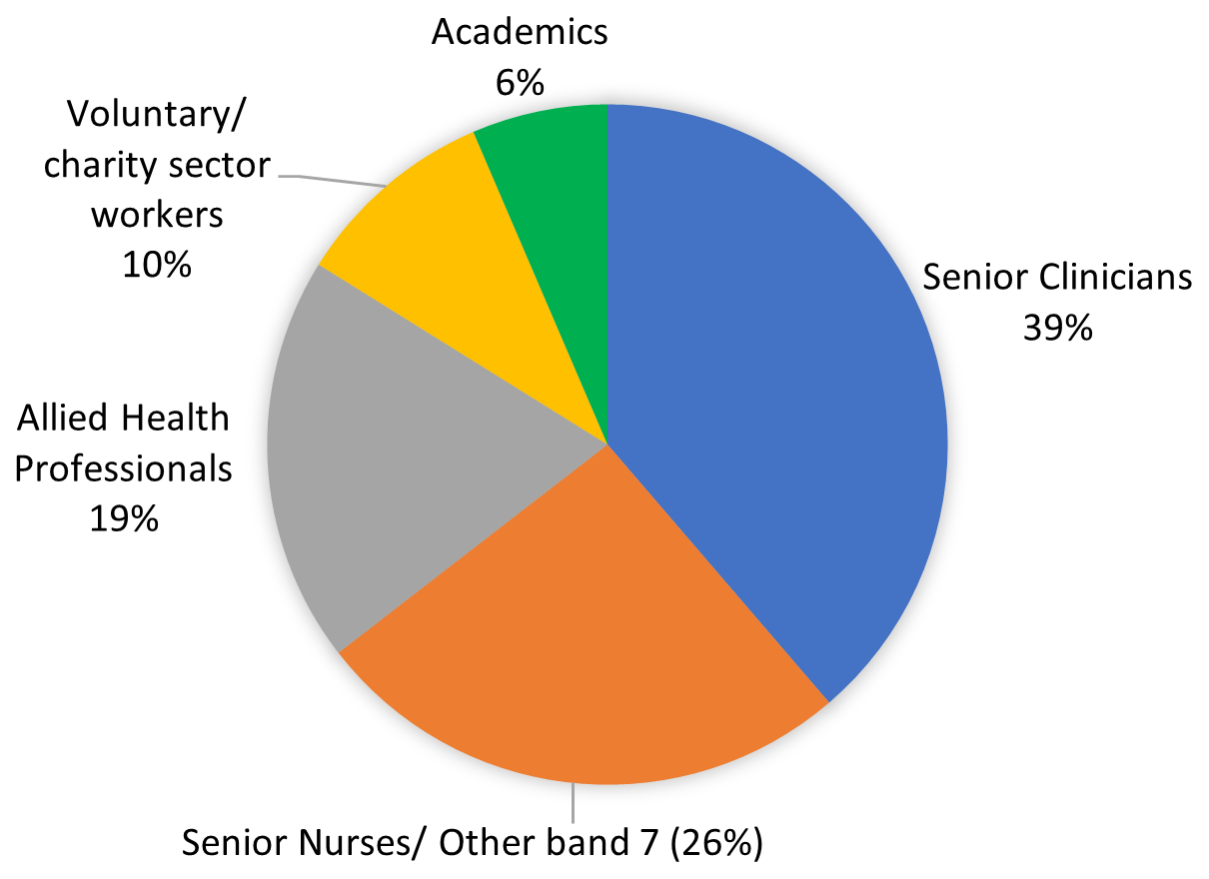
Professions of stakeholder members (n=31).

The stakeholder group consisted of 31 attendees, 12 (39%) of whom were senior clinicians (10 doctors and 2 consultant nurses; Figure 2). There were also senior nurses (26%), allied health professionals (19%), voluntary sector workers (10%) and academics (6%) ( Figure 2).

Including the Project Steering Group, there were 18 males and 25 females, 30 White (British/Irish), 6 Asian or Asian British, 2 Black, Black British, Caribbean or African and 5 White – other.

**Figure 3.**
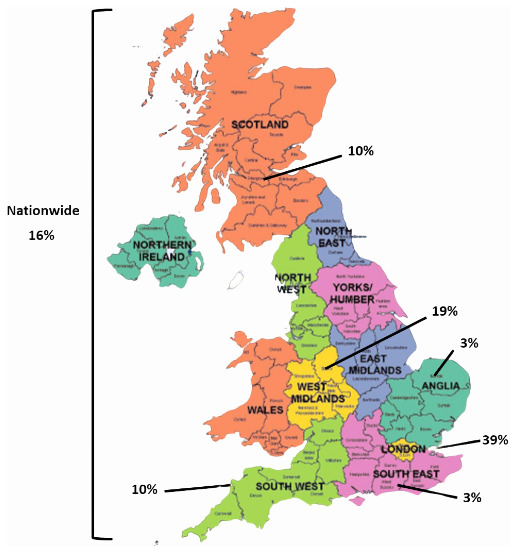
Location of institutions represented in the stakeholder group (n=31). This figure was created with mapchart.net (CC-BY licence).

Nearly 40% of attendees were from institutions based in London. West Midlands had the second biggest representation accounting for 19% of institutions, followed by nationwide representation (16%). Scotland and the Southwest accounted for 10% each, while the East and Southeast accounted for 3% each ( Figure 3).

### 3.5. Outcomes

***3.5.1. Education and awareness.*** One of the main topics that came up in all discussions was the relationship between stigma and lack of education. There was consensus regarding the knowledge gap on the harms of drinking among the wider public, as well a lack of awareness on where and how to seek help. This leads to a reluctance to undergo diagnostic assessment not only due to the fear of a negative result but also the fear that a good result would be perceived as a “green light” to continue drinking. Stakeholders suggested that public education around the wider harms of alcohol should start at school and should use people with lived experience to help deliver it. However, a public health campaign to increase alcohol-related knowledge of the general public would also be useful.

Another issue that emerged in our stakeholder discussions was the misconception that people who are alcohol dependant are not able to understand information and make decisions. This leads to a paternalistic attitude from the healthcare and community services that assume they will not be interested or able to participate in research and so they are often not even approached. Stakeholders reported that the less specialised the HCP, the more judgmental they seem to be towards people with ArLD, whereas people working in hepatology services seem to be more empathetic and less stigmatising. However, PPIE representatives have faced stigmatising behaviour from all types of HCPs ranging from the GP receptionist to the liver unit nurse.

Another topic that was discussed was the gap in ArLD behavioural research. Most ArLD research is liver based clinical trials and fail to address the reasons behind the drinking. This will be further explored in the “Access” section.

***3.5.2. Access.*** Accessibility was considered one of the main barriers to participating in research. Attendees felt that people with ArLD are given less chances to be involved in research than people without ArLD or with other chronic conditions. This stems from several factors, including that most HCPs tend to assume people with alcohol problems are less likely to engage in research. Patients linked with less research-active sites also have less opportunities. Likewise, people whose drinking is currently considered low risk or people who are admitted to hospital with mild liver disease seem to face more challenges in accessing research studies. In fact, most clinical trials include people who have end stage liver disease. There is a motivation to create studies that can help people who are the sickest, overlooking people who drink at less harmful levels. This inhibits pharmaceutical companies from investing in ArLD studies as people who are very unwell at the start of a study have a lower chance of a positive outcome.

Another issue raised was the inconsistency across NHS Trusts. Expertise is not the same throughout the country which creates inequalities in access to care and research. There needs to be national cohesion for standardised care as well as improved communication to avoid duplication of studies and programmes happening across charities and organisations.

The disconnect between liver and mental health services was raised as one of the main problems in treating and engaging people with ArLD. Mental health disorders are more common in people with alcohol problems than the general population, but these are neither proactively acknowledged nor managed. The current approach when people present with symptoms of liver disease is to treat the patient from a hepatology point of view and discharge them with little information and few tools on how to seek help to reduce harmful alcohol use or to address any concurrent mental health problems, which may perpetuate ongoing alcohol use. In some areas, access to mental health services may be limited to people who are not actively drinking alcohol. Consequently, people resort to drinking more instead of getting the support they need and opportunities to help them are missed. There is a pressing need to understand how to recognise and treat mental health issues in people with ArLD.

The issue with funding was also considered. There are currently few social workers working with people with ArLD in hospitals and there are not enough Alcohol Liaison Nurses in the NHS. This contributes to discontinuity in care after discharge and, in many cases, re-admissions.

***3.5.3. Language.*** Language is closely linked to stigma. The terminology around alcohol is not only stigmatising but also has a permanence to it. People who have alcohol problems are classed as “alcoholic”, which is associated with prejudice and negative stereotypes. On the other hand, when people recover from alcohol addiction they are classified as a “recovering alcoholic” and therefore the stigma stays attached to them even after they stop alcohol consumption. The name of the disease containing the aetiology is another source of stigmatisation. Unlike other diseases for which the cause may also be related to lifestyle choices, such as lung cancer (not smoking-related lung cancer) or myocardial infarction (not lifestyle-induced myocardial infarction), ArLD seems to always carry that judgment. While the disease aetiology is important to guide treatment, it is not necessary to label a patient with ArLD permanently.

Jargon and scientific language around ArLD also create barriers. Most people don’t know there are liver specialist teams called ‘Hepatology’ in the hospital. Many don’t understand medical terms like jaundice and ascites. Due to low health literacy in the UK population, language needs to be simplified to be more inclusive.

In addition, alcohol-containing products often say “drink responsibly” but provide little information such as clear content labelling (as is required with food products), placing the responsibility solely on the consumer without telling them how much of their product is safe to drink. This invariably hinders people from realising their drinking habits are harmful until they become unwell.

***3.5.4. Culture.*** The alcohol culture in the UK was considered extremely detrimental to people with an alcohol problem. There seem to be two opposites of the spectrum: while alcohol consumption is widely accepted, once people ask for help, they face stigmatisation. Since symptoms of alcohol dependency are not typically visible, if people are able to function in society, maintain a job or be sociable, it is assumed they do not have a problem.

Old habits also seem to play a role in harmful drinking. Parents encouraging children to drink because they don’t want to drink alone was addressed. Early education could help young people stop the family drinking cycle and reduce peer pressure. However, most attendees agreed that schools are reluctant to address the topic.

The glamourisation of alcohol consumption emerged several times in discussions. The way alcohol is portrayed in the media and publicised on television, films and advertisements perpetuates the expectation that alcohol is necessary for socialising and an important part of daily life. In general, there was consensus regarding the need for a mentality shift to stop glamourising drinking.

### 3.6. Research priorities

Members of the Project Steering Group reviewed the notes from all stakeholder and PPIE meeting discussions and produced a list of four sub-priorities for each priority area ( Table 2). In the last two stakeholder meetings, attendees were asked to anonymously rank each research priority in order of importance from 1–4 (1 being least important and 4 being most important). Accessibility and education/awareness were the highest ranked with a mean score of 3.3 and 3.1 respectively ( Table 2). For each priority, stakeholders also ranked each sub-priority from 1–4. In the “accessibility” theme, “disconnect between liver and mental health services” was the highest ranked with an average of 4, whereas “preconceptions from HCPs” and “lack of funding” were tied as the second most important sub-priority. “Healthcare professionals understanding” came third with an average of 2.7. In terms of “culture”, “media portrayal and promotion of alcohol” was considered the most important aspect, however this is out of remit for the ARMS-Hub as it is not aligned to the Hub’s expertise. Regarding “language”, the fact that terms around alcohol use are stigmatising and have a permanent character was considered the most important sub-priority.

**Table 2.** Mean scores for themes and sub-themes from stakeholder discussions.

*Theme (n=9)*	Mean score	*Sub-theme*	Mean score
Accessibility	3.3	*Disconnect between liver and mental health services* *Preconceptions from HCPs* *Lack of funding* *Inclusion criteria for services/research*	4 2.2 2.2 1.6
Education and awareness	3.1	Lack of knowledge of alcohol harms Healthcare professionals’ understanding Lack of awareness of what is available Misconceptions from public	3.8 2.7 2.2 1.3
Culture	1.9	Media portrayal and promotion of alcohol Improved access for communities where alcohol is taboo Drinking culture in the UK Workplace culture	3.3 2.8 2.5 1.5
Language	1.7	Stigmatising and permanent Messaging e.g. “drink responsibly” Scientific/complex language Linking condition to cause e.g. alcohol-related liver disease	3.5 2.5 2 2

Members of the Project Steering group generated the following research questions from the highest ranked themes: ‘ *Can we design and implement a diverse educational package for both young people and adults around the wider harms of alcohol?’*, ‘ *Can an educational package, co-designed by people with lived experience and stakeholders, be used to reduce stigma in healthcare professionals?’*, and ‘ *How can mental health services best be integrated into liver services?’*. The Project Steering Group decided to focus on the final research question as this was most aligned with Hub expertise and would likely be in remit for a future liver-themed NIHR funding call.

### 3.7. Research symposium

An in-person research symposium was held to discuss and refine the study proposal on integrating mental health services within liver services. The attendees, consisting of 8 Project Steering Group members, 13 HCPs and 4 people with lived experience, were divided into three sub-groups to discuss the three work packages: 1) evidence mapping and synthesis, 2) co-design of an integrated service, and 3) implementation and evaluation of the service.

The group recommended using multiple methods to accumulate information about previous and current integrated liver and mental health services, including a scoping review, surveys and interviews with service providers within the UK and internationally.

There was discussion around the target population for the integrated service. It should not include inpatients as they already have access to mental health support while in hospital but should focus on secondary care outpatient liver services. The design of the intervention should include addiction specialists to help define conditions and treatments to be provided. Attendees suggested having a pathway for screening and making support individualised according to each person’s needs. To ensure representation, cultural and language barriers must be mitigated. Further co-design with stakeholders and PPIE representatives is needed to design an appropriate service.

A full-scale multicentre implementation and evaluation trial on integration of mental health and liver care would not be appropriate at this stage. Instead, there should be focus on feasibility of implementation and cost-effectiveness evaluation of such a service within the NHS. There was agreement that the most important outcome measure should focus on mental health rather than liver indicators, and include satisfaction, alcohol use, prescriptions, admissions, and engagement with services.

There are plans to continue work on this proposal with stakeholders and PPIE representatives in preparation for a future NIHR liver-themed funding call.

## 4. Discussion

Review of the discussions at stakeholder and PPIE group meetings revealed the breadth of experience and opinions on the topic of stigma in ArLD. PPIE representatives drew from their personal experience of stigma to help the group identify the core issues that need to be addressed. These were built on in stakeholder meetings to help define the issues in more detail. Through these discussions we elucidated the main challenges to ArLD research in the UK: the lack of public knowledge about the harms of drinking, the barriers in accessing research and healthcare services, stigmatising language surrounding alcohol use and liver disease, and the detrimental effects of the UK's alcohol culture. Stakeholders prioritised the importance of addressing accessibility and education, with a focus on integrating mental health services within liver services.

This project has demonstrated that research on ArLD need not be confined to region, service or specialty. We drew together an interdisciplinary team representing different professions and services from across the UK. ARMS-Hub will continue to support the enthusiasm and development of this group through the mentorship network.

To our knowledge, this is the first time that a large number of people with lived experience of ArLD from under-represented groups has contributed to research as PPIE representatives. We succeeded in involving people with active alcohol use disorder and people from lower socioeconomic areas, with an appropriate gender balance, representative of ArLD in the UK.1 Despite our efforts to engage with ethnic communities, we only succeeded in involving two people from ethnic minorities with lived experience of ArLD in our PPIE group. We held a variety of meetings in different formats (virtual and in-person) to support access to all people with ArLD. However, we acknowledge that barriers to participation remain: these include poor access to ethnic communities and non-English speakers, and support to participate for those not engaging with alcohol services. Achieving full inclusivity of people with ArLD is still a challenge.

## 5. Conclusions

In conclusion, ARMS-Hub has successfully achieved its objectives. Firstly, we have created an interdisciplinary hub of experts from through the UK interested in ArLD. Secondly, we have engaged with stakeholders and people with lived experience to prioritise research areas around stigma in ArLD. Thirdly, we have brought together an enduring mentorship network that will continue to support each other to enable capacity building through personal and project development. Finally, we have developed the outline of a future proposal to tackle our highest priority research question.

## Data Availability

The full raw underlying data are not available as the meeting transcripts have been deleted as per the study data management plan. However, a collection of written notes made by the ARMS-Hub team during virtual and in-person meetings are available. All queries about the data and methods should be directed to the Chief Investigator Dr Ashwin Dhanda. Open Science Framework; An alcohol-related liver disease multi-stakeholder hub (ARMS-Hub) to enhance research activity in underserved communities in the UK. https://doi.org/10.17605/OSF.IO/ZQB4P ^16^. This project contains the following underlying data: Summarised notes made by researchers (Dhanda, Callaghan and Castro) from stakeholder and PPIE meetings. Open Science Framework; An alcohol-related liver disease multi-stakeholder hub (ARMS-Hub) to enhance research activity in underserved communities in the UK. https://doi.org/10.17605/OSF.IO/ZQB4P ^16^ This project contains the following extended data: Meeting prompts for discussion Example consent and data collection form HRA decisions tool results ( https://osf.io/hbt3w/) Data are available under the terms of the Creative Commons Attribution 4.0 International license (CC-BY 4.0). All other data associated with this project is contained within this manuscript.
