# Supplementary material for: An alcohol-related liver disease multi-stakeholder hub (ARMS-Hub) to enhance research activity in underserved communities in the UK
Source: NIHR Open Res. 2024 Oct 29;4:41. Originally published 2024 Jul 15. [Version 2] doi: 10.3310/nihropenres.13598.2 (PMC11561381; doi:10.3310/nihropenres.13598.2)
Supplement: Peer review history

# Reviewer report: Reviewer response for version 2

**DOI:** 10.3310/nihropenres.14985.r33292

**Published:** 2024-11-13

**Authors:** Hayley Treloar Padovano

**Affiliations:** 1 Brown University, Providence, Rhode Island, USA

Thank you for your thoughtful response to review. I have no further comments.

Is the work clearly and accurately presented and does it cite the current literature?

Yes

If applicable, is the statistical analysis and its interpretation appropriate?

Yes

Are all the source data underlying the results available to ensure full reproducibility?

Yes

Is the study design appropriate and is the work technically sound?

Yes

Are the conclusions drawn adequately supported by the results?

Yes

Are sufficient details of methods and analysis provided to allow replication by others?

No

Reviewer Expertise:

I am an Associate Professor of Psychiatry and Human Behavior and Associate Professor of Behavioral and Social Sciences in the Center for Alcohol and Addiction Studies of Brown University in Providence, Rhode Island. I serve as co-chair of a training-focused Diversity, Equity, Inclusion, and Belonging committee in my primary department, and I am an advisory board member for the Office of Women in Medicine and Science of Brown University Division of BioMed and School of Public Health. I am a licensed psychologist and addiction scientist leading a research program that seeks to improve screening, brief intervention, and referral to treatment/prevention for steatotic liver disease, in partnership with Clinica Esperanza / Hope Clinic. Our team aims to identify and intervene for liver damage from behavioral etiologies at earlier stages. We focus on multilevel determinants of wellness and take action to support inclusivity through addressing barriers to research representation.

I confirm that I have read this submission and believe that I have an appropriate level of expertise to confirm that it is of an acceptable scientific standard.

# Reviewer report: Reviewer response for version 1

**DOI:** 10.3310/nihropenres.14761.r32812

**Published:** 2024-10-09

**Authors:** Hayley Treloar Padovano

**Affiliations:** 1 Brown University, Providence, Rhode Island, USA

Alcohol-associated liver disease has surpassed other causes of liver-death worldwide. Whereas there have been breakthrough treatments for steatotic liver disease from viral causes, alcohol-associated liver disease (ALD) morbidity and mortality rates remain high and rising, particularly among populations that are underrepresented in research and underresourced. This report describes an effort to create interdisciplinary partnerships to address lack of proper prevention and care for ALD. Building from existing ALD networks, this initiative, i.e., the ARMS-Hub, engaged health workers and people with lived experience or their caregivers. It was initiated by a core team of varied stakeholders which were primarily hepatologists (5), as well as a person with lived experience, a nurse, a dietitian, an addiction psychiatrist, a statistician, a research design director, and a qualitative researcher. The ARMS-Hub represents a highly important step toward decreasing morbidity and mortality rates for chronic steatotic liver disease. Key discussion points were the barriers to public knowledge about the harms of alcohol consumption on health, such as media influence and sociocultural facilitators of drinking. Research representation is also limited by the focus of key stakeholders on end-stage liver disease rather than identifying and intervening on behavioral precursors in earlier stages of steatosis and fibrosis where the affected individuals may not be aware of silently progressing liver damage.

 The importance of integrated care is a main theme in this report and a high priority that would be hard to overpromote. The medical complexity of end-stage alcohol-associated liver disease will only increase if rates of heavy drinking continue to rise, particularly among those who are already less likely to receive alcohol-specific care, such as women, individuals from underresourced communities, and healthcare workers themselves. Less than 90% of those who could use alcohol-related services receive it, and the discouraging proportions of those given treatment referrals are the same in the liver clinic as they are in primary care clinic. There were some important issues of reach and disparities in receipt of specialty care at earlier disease stages that were not touched on in this report and may be addressed in future reports or ARMS-Hub iterations. Disparities in alcohol-specific treatment referrals for women and indivdiuals from underresourced communities or of specific ethnicities are noted in the background but not a focus of this report. Adding to concern, women are more likely to be diagnosed with advanced, alcohol-associated liver disease at later stages than men and ciswomen are at greater biological risk of developing liver disease at matched drinking levels and body sizes, theoretically due to differences in body composition and alcohol metabolism. The onus of responsibility for receiving appropriate care at earlier stages cannot continue to be skewed toward the persons who are underresourced and in need of care. Efforts such as ARMS-Hub are taking important steps toward imploring healthcare providers and researchers in liver- and alcohol-focused fields to expand our reach.

 Active efforts to be inclusive of individuals with lived experience from diverse backgrounds and underrepresented groups, such as through engaging community leaders, are an ARMS-Hub strength. These strategies did not appear to be particularly effective, however, and ongoing attention to barriers to inclusivity and diversity in the patient and public involvement and engagement (PPIE) group is needed. Toward that goal, the criteria for inclusion may be better described in this report. For example, were particiants required to speak and read English? If so, why? Any other criteria for participation in either the PPIE or stakeholder groups may also be described. The demographic information for gender and ethnicity of the stakeholder participants is not reported. This must be corrected and is the reason for reserving approval for this report. Had it been collected, other descriptive information for particupants, such as sexual and gender identity, would have also enriched this report. It is remarkable that nearly half of the in-person group and one-third of the virtual group declined to indicate their liver-health status or the status of the person cared for. These numbers speak. 

 New partnerships with policymakers and nonprofit organizations serving individuals from underresourced communities will be imperative in future iterations of the ARMS-Hub. Progress may also be faster if the decision-making bodies writing the guidelines and consulting with funding agencies were more inclusive of other disciplines in addition to medicine, such as psychological, social, or behavioral sciences and public health. It is noteworthy that the representative organizations for stakeholders in the ARMS-Hub appeared to draw heavily from liver-focused subspecialties in medical fields. The report concluded that mental health services must be better integrated. It might be worth taking the additional step to call for bringing alcohol-specific behavioral health services into liver healthcare spaces. In any case, it is essential that persons doing this work on a daily basis are included in future conversations and that people with lived experience continue to be viewed as experts of their own lives. The decision-makers and those writing future guidelines need to be reflective of the full range of expertise necessary to address this immense unmet need.

 Major strengths and future priorities summarized above also follow in numbered comments that specifically point to sections of the ARMS-Hub report. Many thanks for the opportunity to comment and for the ARMS-Hub members who are leading this charge which is both challenging and life saving.

  

1. Directive questions were asked of focus groups rather than more open-ended phrasing. For example, “Does integration of mental health services improve quality of life and clinical outcomes for people with alcohol-associated liver disease?” may have been phrased “How might integration of mental health services influence quality of life and clinical outcomes for people with ALD?” A rationale for the more directive phrasing and overall approach is not provided.
2. Basic demographic information for the gender and ethnicity of stakeholders needs to be described. If available, it may also be of interest to readers to report the descriptive information of ARMS-Hub members regarding percent effort or time engaging in clinical practice vs. research and whether the focus of their work is in alcohol-related treatment and research fields vs. other substance-use or mental-health related spaces.
3. Ranking data described with mean score may also include a standard deviation to get a sense of the spread of rankings, and/or counts within rankings provided in full in a supplement. (2.7)
4. Meetings with recorded PPIE and stakeholder discussions were not considered qualitative data that could be analyzed via a structured approach. The rationale for this approach is not provided. Future iterations may consider best practices for collecting and analyzing the data from these groups, making the most of the participants’ time and minimizing bias in reporting results. (2.7)
5. What are potential barriers to participation in both the virtual and in-person PPIE groups? There were considerable numbers who expressed interest who did not then participate. This may highlight the importance of identifying poor liver health earlier in the progression, prior to significant medical complications and barriers to care and research representation.
6. How do the reported demographics of participants compare to those of the target communities?
7. With regard to the potential “green light” to continue drinking for a screening that does not indicate steatosis or fibrosis, this can be mitigated via framing the result as a relief to know that, even with these significant behavioral risk factors, the person has not yet developed liver disease, and, further, that change in these habits has time to avoid the progression towards steatotic liver disease. (3.5.1). It is understood that the results are reporting the findings from these groups, but it is important to make sure that these findings do not continue to perpetuate these beliefs without additional corrective context.
8. An excellent point is made regarding research representation for individuals with steatotic liver disease in the earlier stages. The rationale provided focuses mainly on the inability of the person to access research studies, rather than potential gatekeeping and bias. Those in the decision-making circles (e.g., experts in societies writing the recommendations) appear to be primarily hepatologists whose research focuses on end-stage liver disease. Partnership with individuals from other disciplines, e.g., public health scientists, psychologists, social work, would broaden viewpoints. It would be interesting to gather data on the number of local studies/clinics of individuals who drink heavily who may not know that they have early-stage liver-health issues.
9. Were there differences in results and viewpoints between the PPIE and stakeholder groups? Even if not able to be statistically defined, it would be interesting to compare even descriptively the potential differences in opinions or proposed best practices between academic circles and PPIE. For example, were issues around language and stigmatizing terms raised by people with lived experience or stakeholders?
10. Were there included individuals who could have been included in either the stakeholder or the PPIE group? It is presented as though these two are necessarily distinct, but rates of alcohol consumption among healthcare workers are high, and there is known issues to seeking help out of fear of work-related consequences.
11. Was English language a requirement to participation in these groups? If so, what was the rationale for this?

Is the work clearly and accurately presented and does it cite the current literature?

Yes

If applicable, is the statistical analysis and its interpretation appropriate?

Yes

Are all the source data underlying the results available to ensure full reproducibility?

Yes

Is the study design appropriate and is the work technically sound?

Yes

Are the conclusions drawn adequately supported by the results?

Yes

Are sufficient details of methods and analysis provided to allow replication by others?

No

Reviewer Expertise:

I am an Associate Professor of Psychiatry and Human Behavior and Associate Professor of Behavioral and Social Sciences in the Center for Alcohol and Addiction Studies of Brown University in Providence, Rhode Island. I serve as co-chair of a training-focused Diversity, Equity, Inclusion, and Belonging committee in my primary department, and I am an advisory board member for the Office of Women in Medicine and Science of Brown University Division of BioMed and School of Public Health. I am a licensed psychologist and addiction scientist leading a research program that seeks to improve screening, brief intervention, and referral to treatment/prevention for steatotic liver disease, in partnership with Clinica Esperanza / Hope Clinic. Our team aims to identify and intervene for liver damage from behavioral etiologies at earlier stages. We focus on multilevel determinants of wellness and take action to support inclusivity through addressing barriers to research representation.

I confirm that I have read this submission and believe that I have an appropriate level of expertise to confirm that it is of an acceptable scientific standard, however I have significant reservations, as outlined above.

# Response

**Published:** 2024-10-25

**Authors:** Ashwin Dhanda

**Affiliations:** University of Plymouth, Plymouth, England, UK

Thank you for your supportive and insightful comments on our manuscript.

 The ARMS-Hub stakeholder group was successful in involving a diverse interdisciplinary group of experts by experience and training. Although we had over-representation of hepatologists, we believe this is justified as our focus was on alcohol-related liver disease (ArLD) rather than alcohol use disorder. We did obtain representation from people with lived experience of all stages of ArLD from early steatosis (32% in the in-person PPIE group) to decompensated cirrhosis (32% in the in-person PPIE group) so were not solely focused on end-stage liver disease.

 We agree that our efforts to achieve inclusivity fell short of our ambitions. We did involve people from the full range of socioeconomic indices and had an appropriate gender balance but we only had two people of Asian heritage with lived experience of ArLD. We have added this as a limitation to the discussion section. We included options for people to describe themselves as non-binary/non-conforming, transgender or other but none selected these. We did not report gender or ethnicity of our stakeholder group, which has now been added in section 3.4. Including the Project Steering Group, there were 18 males and 25 females, 30 White (British/Irish), 6 Asian or Asian British, 2 Black, Black British, Caribbean or African and 5 White – other.

 In response to your specific points:

1. The directive questions were generated by the Project Steering Group after review of the information obtained from the five stakeholder meetings and were not used as the basis of discussions in the group meetings. The question “Does integration of mental health services improve quality of life and clinical outcomes for people with alcohol-associated liver disease?” was the subject of the group work in the research symposium. The question itself was not discussed but elements of a research proposal to address this question were considered (evidence synthesis, co-design methods and implementation and evaluation). This has been described in section 3.6.
2. Basic demographic information for gender and ethnicity of stakeholders has now been included in section 3.4. Unfortunately, we did not collect data on stakeholders’ clinical or research allocation and focus of work.
3. Ranking of research themes and sub-themes was performed using free online software, which does not provide individual participant rank scores. Therefore, we are unable to provide raw scores or standard deviations for mean scores.
4. This study was not considered research and therefore a less analytical approach to data reporting was taken. Data were summarised by hand rather than being transcribed or thematically analysed in line with NIHR guidance (Public Involvement Development Partnership report 2019). Section 2.7 has been updated accordingly.
5. Fourteen people registered for the virtual PPIE meetings and nine attended at least one meeting. Reasons for drop outs were not obtained. We designed the PPIE meetings with a flexible approach in mind to support people that might find it difficult to participate otherwise. Our in-person PPIE meetings used an opportunistic sample of people who were attending a community alcohol service on a particular day. Therefore, accessibility to the meeting was not an issue. We scheduled several virtual PPIE meetings at different times of the day to permit people to attend meetings when convenient. However, we acknowledge that these meetings were held during working hours and may have had different attendance if held in evenings or weekends. Despite our best efforts, barriers to participation remain: these include poor access to ethnic communities and support to participate for those not engaging with alcohol services. We have added this as a limitation in section 4.
6. In the UK, ArLD disproportionately affects those of lower socioeconomic status. It remains a male predominant condition but prevalence among women is increasing (Office of Health Improvement and Disparities, Alcohol Profile: https://fingertips.phe.org.uk/profile/local-alcohol-profiles/data#page/1). Accurate epidemiological data on ethnicity is not available in the UK but approximately 90% of people presenting to hospital with ArLD are White English/Irish. One study in Scotland showed that more men than women had hospital admissions or death from ArLD (27,623 vs 24,285) and 1,106 (2.1%) were from ethnic minorities (Bhala et al, Alcohol and Alcoholism 2016; doi: 10.1093/alcalc/agw018). Our PPIE group has captured the range of socioeconomic status and achieved a representative gender balance. As described above, representation from ethnic minorities could be improved. This is now included in section 4.
7. We acknowledge that work is underway to evaluate methods of framing fibrosis risk scores as a behaviour change intervention.
8. We agree that it is paramount to tackle early ArLD and that further interdisciplinary research efforts are needed in this area.
9. This level of detail was not recorded in the meeting notes but would be interesting to understand in future work.
10. We agree that there is overlap between stakeholders and PPIE members. There were at least three stakeholder/PPIE members who were both experts by training and experience. This enabled them to provide a unique perspective on the topic. We did not ask stakeholders to provide information about their alcohol use.
11. English language proficiency was not a requirement for participation. However, we did not proactively seek representation from non-English speakers and none expressed an interest in participating. We need to establish improved methods to engage non-English speakers and people from ethnic minorities in future studies. We have included this as a limitation in section 4.

# Reviewer report: Reviewer response for version 1

**DOI:** 10.3310/nihropenres.14761.r32629

**Published:** 2024-08-29

**Authors:** Cathy Beresford

**Affiliations:** 1 Bournemouth University, Poole, England, UK

I was pleased to review this paper regarding the alcohol-related liver disease multi-stakeholder hub (ARMS-Hub) to enhance research activity in underserved communities in the UK. The article provides a thorough and well-explained overview of the project, which aimed to address the significant gap in research on Alcohol-related Liver Disease (ArLD) by building interdisciplinary partnerships, exploring stigma, involving people with lived experience, and developing targeted research proposals. The collaborative nature of the work is clearly articulated, with transparency regarding who was involved, and the aims and objectives are expressed clearly. The methods used to achieve these objectives are also apparent, demonstrating a strong commitment to addressing the challenges in ArLD research. Through these collaborative efforts, the ARMS-Hub identified major research areas, including the disconnect between mental health and liver services, and established a mentorship programme to support emerging researchers.

 Where you have stated that you succeeded in involving people from ethnic minorities (section 4), I suggest that you might expand on this slightly to acknowledge that ideally, more diverse representation in terms of ethnicity would have been preferred. I appreciate that you have made concerted efforts to reach a range of people (as explained under section 2.3), but in section 3.3.1., you note that one person from the PPIE virtual group was Asian or Asian British and the rest were White. It is reasonable to acknowledge that it is challenging to achieve full inclusivity, even when appropriate efforts have been made.

 I do not have any further recommendations for changes, as this is an excellent and thorough account of the project. I look forward to reading future work from the ARMS-Hub.

Is the work clearly and accurately presented and does it cite the current literature?

Yes

If applicable, is the statistical analysis and its interpretation appropriate?

Not applicable

Are all the source data underlying the results available to ensure full reproducibility?

Yes

Is the study design appropriate and is the work technically sound?

Yes

Are the conclusions drawn adequately supported by the results?

Partly

Are sufficient details of methods and analysis provided to allow replication by others?

Yes

Reviewer Expertise:

Experiences of care in advanced liver disease. Public involvement. Care in long-term conditions. Qualitative research.

I confirm that I have read this submission and believe that I have an appropriate level of expertise to confirm that it is of an acceptable scientific standard.

# Response

**Published:** 2024-10-25

**Authors:** Ashwin Dhanda

**Affiliations:** University of Plymouth, Plymouth, England, UK

Thank you for the supportive comments.

 We have amended section 4 to more clearly describe that ethnic diversity was not fully captured in this work.
